# Iron Deficiency Anemia in Inflammatory Bowel Disease: What Do We Know?

**DOI:** 10.3389/fmed.2021.686778

**Published:** 2021-07-01

**Authors:** Tamás Resál, Klaudia Farkas, Tamás Molnár

**Affiliations:** Gastroenterology Unit, Department of Medicine, University of Szeged, Szeged, Hungary

**Keywords:** inflammatory bowel disease, iron deficiency anemia, iron supplementation, anemia, parenteral iron supplementation, oral iron supplementation

## Abstract

One of the most common extraintestinal manifestations of inflammatory bowel disease is iron deficiency anemia. It is often an untreated condition that significantly impairs patients' quality of life and elevates mortality and morbidity. Although it is often accompanied by mild symptoms (e.g., fatigue, lethargy), it can provoke severe health conditions, such as dyspnea, palpitation, angina, and mental disorders, and increases hospitalization and mortality rate as well. As anemia develops through several pathomechanisms, such as occult bleeding, chronic inflammation, and medicines (e.g., methotrexate), treating anemia effectively requires to manage the underlying pathological changes as well. Based on international publications and data, it is a frequent condition and more frequent in pediatrics. According to Goodhand et al., iron deficiency is present in more than 60% of children, whereas only 14% of them received oral iron therapy. Compared to adult patients, 22% have iron deficiency, and 48% of them received oral and 41% intravenous iron therapy. Miller et al. also highlighted that among young patients iron deficiency anemia is a frequent condition, as almost 50% of the patients were anemic in their cohort. European Crohn's and Colitis Organisation's statements are clear regarding the diagnosis of iron deficiency anemia, and the iron supplementation as well. Third-generation parenteral iron supplementations seem to be safer and more effective than oral iron pills. Oral iron in many cases cannot replace the iron homeostasis as well; furthermore, it can provoke dysbiosis, which can potentially lead to relapse. As a result, we claim that both oral and parenteral should be used more frequently; furthermore, intravenous iron could replace oral medicines as well in certain cases. Despite the fact that iron deficiency anemia is examined by many aspects, further questions can be raised. Can it imply underlying pathological lesions? Are both oral and intravenous iron therapy safe and effective? When and how are they used? We demand that more studies should be conducted regarding these issues.

## Introduction

Inflammatory bowel disease [IBD: Crohn disease (CD), ulcerative colitis (UC)] is a chronic, immune-mediated disease that impairs patients' quality of life (QoL), and it is associated with many comorbidities. One of the most common concomitant diseases is iron deficiency anemia (IDA), which also worsens the condition of patients and mostly remains untreated (1). It can occur at any stage of IBD and can be the first symptom of the disease as well. It is often associated with frequent chronic activity, but can also be encountered without clinical signs of activity. In such cases, the IDA diagnosis raises the possibility of asymptomatic, subclinically occurring inflammation and mucosal damage in the presence of long-term chronic activity that damages the condition and function of the intestine (1).

### Frequency of IDA

At the time of the diagnosis, the prevalence of IDA in patients younger than 18 years is approximately 41–75% (2), whereas in adult patients it is also high; however, it varies with wide ranges, from 6 to 74% (3). According to a Swedish study, conducted by Sjöberg et al., the prevalence of IDA is almost twice as high in children (55%) as in adult patients (27%) at the time of the diagnosis. Furthermore, they found significant difference as well in the prevalence of IDA among patients with CD, compared to UC, following the first year after the diagnosis. They also found that anemia in CD was more common in colonic engagement, and in UC, extensive inflammation increased the prevalence (4). Eriksson et al. conducted a study to assess the incidence, prevalence, and clinical outcome of anemia in IBD, comparing CD and UC, and they found as well that CD is associated with higher prevalence and a worse outcome regarding the resolution of anemia (5).

### Symptoms and Clinical Role

Generally speaking, it should be highlighted that patients with IDA claim to have decreased QoL. In a Spanish study, conducted by García-López et al., it was found that treating IDA improves the QoL, regardless of the symptoms of IBD (6).

As iron plays key role in the function of many cells (e.g., erythrocyte, macrophage), cellular proteins, and enzymes (e.g., cytochromes, myoglobin), the symptoms of IDA vary over a wide range (7, 8). Key symptoms, such as shortness of breath, palpitation, tachycardia, and even angina, occur because of the hypoxemia. As a result of the decreased blood oxygen level, there is a compensatory decrease in intestinal blood flow, which may cause motility disorder, malabsorption, nausea, weight loss, and abdominal pain. Central hypoxia may lead to headache, vertigo, and lethargy, as well as cognitive impairment, and several studies proved that normalizing anemia improves cognitive functions (9–12) (Table 1).

**Table 1:** Symptoms of iron deficiency anemia.

Nervous system	Headache, lethargy, vertigo, syncope, cognitive impairment, depression
Cardiovascular system	Palpitation, tachycardia, hypotension, angina, ischemic electrocardiographic signs, cardiac failure
Respiratory system	Shortness of breath
Skin	Paleness, alopecia, cold intolerance
Gastrointestinal symptoms	Anorexia, nausea, motility disturbances, angular stomatitis, glossitis (Plummer–Vinson syndrome)
Immune system	Disorder of the innate and adaptive immune system
Urogenital symptoms	Decreased libido, menstrual disorders
General symptoms	Decreased quality of life, lower physical activity

Michailidou et al. compared the risk of postoperative complications between anemic and nonanemic patients. In their study consisting of more than 15,000 people, it was found that patients with anemia were more likely to have postoperative complications (e.g., morbidity and mortality rate; undesirable cardiovascular, renal, pulmonary, and wound healing complications; postoperative sepsis and shock) (13).

### Etiology of Anemia in IBD

The most common causes of anemia in IBD are IDA, chronic inflammation, and anemia of mixed origins, whereas B_12_ deficiency and folic acid deficiency (mostly due to medications) belong to the less common causes. In addition, it may also occur because of hemolysis, myelodysplastic syndrome/medication-induced aplasia, protein starvation, and liver disease (e.g., primary sclerosing cholangitis) (14, 15) (Table 2).

**Table 2:** Ethiology of anemia in IBD.

Most common causea of anemia in IBD	-Iron deficiency anemia -Anemia of chronic inflammation -Anemia of mixed origins
Less common causes	-Folic acid/B_12_ deficiency
Rare causes	-Hemolysis -Myelodysplastic syndrome -Aplasia -Protein starvation -Liver disease

### Pathophysiology of IDA

In IBD, the IDA can develop through several pathomechanisms (16):

I. Intestinal mucosal damage resulting in occult, chronic blood loss

II. Chronic inflammation

a) Reduced iron-absorbing capacity of enterocytes
b) Iron is trapped in macrophages
c) Inhibition of the erythropoietin and the differentiation/proliferation of the erythroid progenitor cells

Cytokines and acute-phase proteins cause changes in iron homeostasis during inflammation. Hepcidin plays the central role in the regulatory process. It is an antimicrobial protein, produced by the liver in case of iron surplus and in inflammation, triggered by interleukin 6 and lipopolysaccharides. Hepcidin binds to the iron-transporting ferroportin receptor and degrades it, which results in decreased iron transport from the enterocytes to the circulation, and causes retention of the iron in the monocytes/macrophages; these processes are enhanced by anti–tumor necrosis factor α. In addition, hepcidin reduces the absorption of the Fe^2+^ from the duodenum, through the inhibition of the DMT1 (divalent metal transporter 1) (17).

Transferrin is the main iron carrier protein, and during inflammation, acute-phase proteins (e.g., α-1 antitrypsin) bind to transferrin receptors and inhibit the iron uptake in the erythroid progenitors cells, resulting in reduced differentiation and proliferation (18).

### Diagnosis/Differential Diagnosis in Anemia

Anemia and iron homeostasis should be monitored regularly in IBD (depending on activity and the type of the treatment):

- At the time of diagnosis
- During activity—every 3 months
- In remission—every 6 to 12 months

Vitamin B_12_ and folic acid should be monitored every year, in case of presence of risk factors (e.g., resection, pouch, extensive ileal disease) every 3–6 months.

The diagnosis of anemia is assessed by the World Health Organization (WHO) diagnostic criteria, depending on the gender and age of the patients (Table 3).

**Table 3:** Pathomechanisms of different type of anemias in IBD.

Iron deficiency anemia	Chronic blood loss Reduced absorption of Fe^2+^ (bowel resection, inflammation) Anorexia
Anemia of chronic disease	Iron retention in monocytes/macrophages Reduced absorption of Fe^2+^ (inflammation) Reduced biological half-life of erythrocytes (e.g., erythrophagocytosis) Inhibition of erythropoiesis
Other origin	Vitamin deficiency (B12, folic acid) Drug-induced bone marrow suppression (methotrexate, azathioprine)

### Differential Diagnosis in Anemia

According to the European Crohn's and Colitis Organization (ECCO) recommendations, the following parameters should be monitored if hemoglobin is below normal (Table 4): erythrocyte count, serum ferritin, C-reactive protein (CRP) concentration, transferrin saturation, reticulocyte count, erythrocyte width distribution, and mean corpuscular volume. Ferritin is an acute-phase protein produced by the liver, and it is responsible for binding and storing iron in the liver, spleen, and reticuloendothelial system. It is reduced in case of iron deficiency and elevated in inflammation. Hence, in determining the cause of anemia in IBD, it is important to assess disease activity based on disease scoring systems (Crohn's Disease Activity Index and Mayo score) and serum CRP and fecal calprotectin levels. In case of inflammation, transferrin saturation helps in differential diagnosis. Transferrin saturation (accepted normal range = 20–45%) is lower in inflammation, liver disease, malignancy, nephrotic syndrome, and anorexia, whereas it is elevated in iron deficiency and pregnancy (19).

**Table 4:** World Health Organization's anemia criteria.

	**Hemoglobin (g/dL)**	**Hematocrit(%)**
Children between 6 months and 5 years	11	33
Children between 5 and 11 years	11.5	34
Children between 12 and 13 years	12	36
Pregnant women	11	36
Women	12	33
Men	13	39

IDA (19):

a) Anemia based on WHO criteria (low hemoglobin and hematocrit). Clinically and endoscopically, no inflammation can be found, CRP level is normal, and serum ferritin is <30 μg/L.
b) Anemia based on WHO criteria. Clinically and/or endoscopically, inflammation can be found, CRP is elevated, and ferritin is <100 μg/L.

Anemia associated with chronic inflammation:

- Anemia based on WHO criteria. Clinically and/or endoscopically, inflammation can be found, CRP is elevated, ferritin is >100 μg/L, and transferrin saturation is <20%.

**Figure 1:**
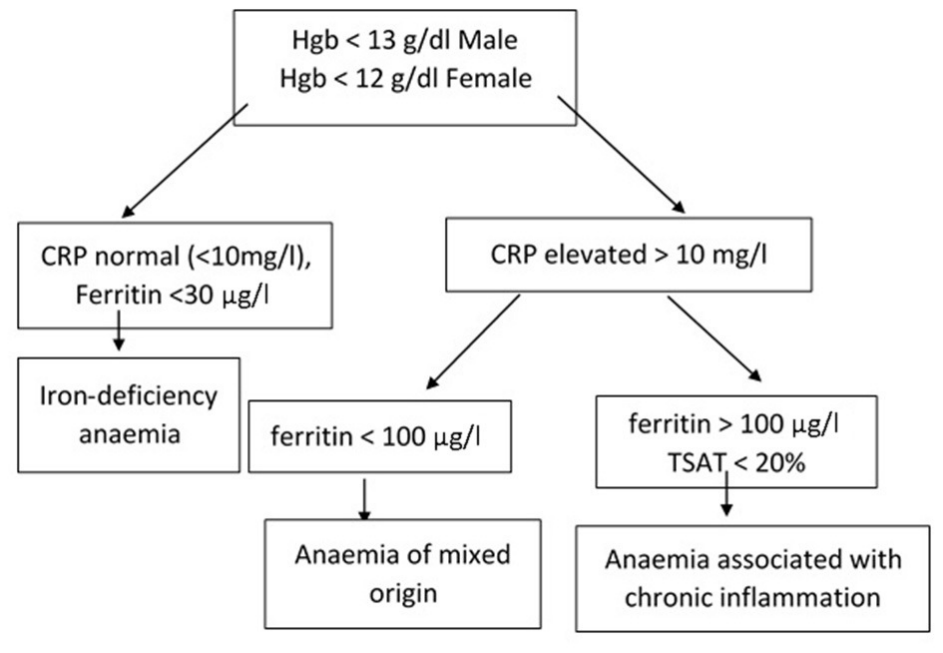
Differential diagnosis of IDA and chronic inflammation–associated iron deficiency.

Anemia of mixed origin:

- Anemia based on WHO criteria. Clinically and/or endoscopically, inflammation can be found, CRP is elevated, and ferritin is between 30 and 100 μg/L (Figure 1).

### Iron Supplementation

Goodhand et al. pointed out how undertreated the IDA is in IBD. In children (88%) and adolescents (83%), the incidence of IDA is much higher compared to that in adults (55%), and only a small proportion of patients received oral (children 13%, adolescents 30%, adults 48%) or parenteral iron supplementation (children 0%, adolescents 30%, adults 41%) (20).

In addition to improving the QoL, the goal of iron supplementation is to normalize hemoglobin, serum ferritin, and transferrin saturation and to refill iron stores (ferritin >100 g/L).

Based on the recommendation of the ECCO (19):

Iron supplementation is recommended to all patients with anemia associated with iron disorder. If iron deficiency exists without anemia, iron supplementation requires consideration of the patient's individual clinical status, as there is no evidence in IBD regarding the efficacy of the treatment (19).

Oral iron supplementation is recommended to every patient with IDA with hemoglobin >10 g/dL in case of remission (no clinical/endoscopic activity, normal CRP level). The recommended oral iron intake is 100 mg/day for adults (higher doses are not recommended), and 2–3 mg/kg body weight per day in children. An acceptable therapeutic response is an increase of 2 g/dL in hemoglobin over 4 weeks. If there is intolerance, adverse effects or unsatisfactory therapeutic response is present, intravenous iron therapy is recommended (19).

Parenteral iron supplementation is recommended as a first choice in IDA in case of active IBD (elevated CRP levels and/or clinically active IBD), or hemoglobin level <10 g/dL, or previous intolerance to iron supplementation is present. If the elevation is <2 g/dL in hemoglobin level after 4 weeks' therapy, it is recommended to complete the treatment with Erythropoietin (EPO) stimulant. The required iron intake is estimated based on the body weight and the hemoglobin, as it is more effective in patients with IBD suffering from iron deficiency than the traditional Ganzoni formula (19) (Table 5).

**Table 5:** Administration of parenteral iron replacement.

**Hemoglobin (g/dL)**	**Body weight <70 kg**	**Body weight ≥70 kg**
10–12 (Female)	1,000 mg	1,500 mg
10–13 (Male)		
7–10	1,500 mg	2,000 mg

Following the resolution of IDA with parenteral iron supplementation, ferritin level is recommended to be maintained above 400 μg/L to prevent short-term recurrence. After successful iron supplementation, patients should be monitored every 3 months in the first year following the correction and every 6–12 months thereafter (including hemoglobin, ferritin, transferrin saturation, CRP). Recurrent anemia may indicate underlying inflammation despite clinical and biochemical remission. The goal of preventive treatment is to keep ferritin and hemoglobin at normal levels. Reinitiation of intravenous iron supplementation is recommended in cases where the ferritin level falls below 100 μg/L or the hemoglobin level is <12–13 g/dL (female/male) (19).

When considering between oral and intravenous iron therapy, the advantages and disadvantages of the therapeutic approaches should be considered as well (21–23).

#### Oral Iron Supplementation

a. Advantages
  Low cost
  Easier to implement in daily practice
  More accessible
  Effective in good intestinal absorption
b. Disadvantages
  Compliance issues
  Certain foods reduce iron absorption (e.g., tea, coffee, dairy products, fiber)
  Certain medications reduce iron absorption
  i. Multivitamin/dietary supplements (Ca^2+^, Zn^2+^, Cu^2+^)
  ii. Antacids, H_2_ blockers, PPI
  iii. Quinolones, tetracyclines
  Dysbiosis
  Dysbiosis induced relapse
  Side effects are more common compared to parenteral iron suppl.
  i. Nausea
  ii. Abdominal pain
  iii. Diarrhea
  iv. Constipation

#### Parenteral Iron Supplementation

a. Advantages
  More effective
  Fast correction of iron homeostasis
  Safe and well tolerated
  Fewer side effects
  Effective in inflammation
  The condition of the mucosa does not influence the efficacy
b. Disadvantages
  Higher cost
  Harder to implement in daily practice
  Potential risk of iron overload
  Potential risk of anaphylaxis
  Possibility of hypophosphatemia

### Parenteral Iron Supplementation

Intravenous iron supplementations consist of an Fe^3+^ core and a carbohydrate layer. The side effect profile, clearance, tolerable dose, and duration of the infusion are dependent on the magnitude of the core and quality of the carbohydrate layer. The different generations of intravenous iron supplementation comprised different carbohydrate layers (24).

- First generation—high-molecular-weight iron dextran
- Second generation—low-molecular-weight iron dextran
  a. Ferrous gluconate
  b. Iron sucrose
- Third generation
  a. Ferumoxytol
  b. Iron carboxymaltose
  c. Iron isomaltoside

The disadvantage of the HMWID is the higher probability of anaphylactic reaction/side effects; because of that, it is advised to use higher-generation products. The representatives of the second generation are more efficient with fewer side effects; however, they are not as stable complexes as the representatives of the third-generation preparations; consequently, they can only be administered in low doses, and so they require frequents visits. Third-generation preparations are much more efficient, with minimal side effect profile; furthermore, they can be implemented easier in the daily practice. These formulations are more stable, so they can be administered in higher doses, resulting in faster correction of the iron homeostasis, and the duration of the infusion is lesser (23, 25).

## Discussion

Anemia and IDA are common consequences of IBD in the developed world. Despite that we know how frequent it is, physicians tend to pay less attention to treat it, even though it affects the course of the disease and heavily reduces the patients' QoL. Although modern medicine knows many facts about the pathophysiology of anemia and IDA, and there are many efficient agents in the therapeutic arsenal, it still raises relevant questions. However, the ECCO's recommendations are clear; we would like to highlight that it should be still a matter of individual judgment, and in certain cases, parenteral iron supplementation should be the choice, instead of oral, because of the side effects. Based on international publications and data, as intravenous iron supplementation tends to be more efficient and safe in IBD, we claim that more studies should be conducted regarding third-generation agents and clarify the boundary line in the recommendations. However, to sum up, we demand that both oral and intravenous iron treatments should be more widespread.

## Author Contributions

All authors listed have made a substantial, direct and intellectual contribution to the work, and approved it for publication.

## Conflict of Interest

KF has received speaker's honoraria from AbbVie, Janssen, Ferring, Takeda, and Goodwill Pharma. TM has received speaker's honoraria from MSD, AbbVie, Egis, Goodwill Pharma, Takeda, Pfizer, and Teva. The remaining author declares that the research was conducted in the absence of any commercial or financial relationships that could be construed as a potential conflict of interest.
